# The Unique Role That WHO Could Play in Implementing Phage Therapy to Combat the Global Antibiotic Resistance Crisis

**DOI:** 10.3389/fmicb.2020.01982

**Published:** 2020-09-03

**Authors:** Alan Fauconnier, Tobi E. Nagel, Charlotte Fauconnier, Gilbert Verbeken, Daniel De Vos, Maia Merabishvili, Jean-Paul Pirnay

**Affiliations:** ^1^Culture In Vivo ASBL, Nivelles, Belgium; ^2^Phages for Global Health, Oakland, CA, United States; ^3^Laboratory for Molecular and Cellular Technology, Queen Astrid Military Hospital, Brussels, Belgium

**Keywords:** phage therapy, World Health Organization, low and middle-income countries, regulatory framework, local production, GMP, database, deep learning

## Introduction

Given the immensity of the global antimicrobial resistance (AMR) crisis, new avenues complementary to traditional antibiotics are urgently needed, particularly in developing countries where 90% of the predicted AMR deaths will occur (O'Neil, 2014). Bacteriophages (also known as phages) are a class of natural antimicrobials that were used, sometimes successfully (Abedon et al., 2011), before chemical antibiotics were discovered, and in recent years phages have been utilized to treat antibiotic-resistant infections (Kortright et al., 2019). Indeed, phage therapy is now undergoing a renaissance in industrialized countries, though their use has been little explored for low-income and lower-middle-income countries (LMICs). Rehabilitation of phage therapy represents both challenges and opportunities, which need to be dealt with at both local and global levels. Because of this, the World Health Organization (WHO) appears uniquely positioned to play a key role in the deployment of this atypical but promising technology as a means to combat antibiotic resistance.

## The Pressing Need in Developing Countries

Phage therapy could be especially valuable for LMICs. In such settings, contaminated food or water, antimicrobial resistance, and also sanitary and hygiene problems are predisposing factors for bacterial infection. Moreover, malnutrition and HIV-mediated immunosuppression make their populations more vulnerable. There is thus an urgent need for supplementary treatment modalities in these areas, calling for the potential of phages to be unleashed in LMICs (Nagel et al., 2016).

As explained by Nagel et al. (2016), phages display several characteristics making them particularly suitable for LMICs. For instance, since phages are highly specific, they preserve commensal flora, which is a significant benefit for malnourished and immunocompromised individuals. In addition, phages can be easily isolated and produced locally using basic technological tools readily available in LMICs. The manufacturing of phages, excluding burden and costs incurred by regulatory and Good Manufacturing Practices (GMP) compliance, is inherently rapid and cost-effective. Also, the extemporaneous use of customized phage therapy medicinal products (PTMPs) could potentially minimize storage issues since cold-chain might not be needed. Phages could be complementary to vaccines since the quick killing activity of phages could buy enough time for the immune system to build a protective response to invading pathogenic bacteria. In addition to direct administration to people, phages can also be used to prevent food contamination (Sulakvelidze, 2013; Moye et al., 2018). Finally, encouraging laboratories and biotech companies to develop new phage products, as part of the response to the immense public health needs of LMICs, could also contribute to the development of new market opportunities.

Accordingly, phage technology should be implemented in LMICs as part of a process aimed at transferring technology to local spheres and community-based management. And because of its public health experience and expertise in LMICs, WHO could serve as a reliable stakeholder, helping to ensure such knowledge transfer. In addition, WHO could play a central role in a global management plan to limit resistance development against phages by following pathogen evolution and diversity in LMICs. In this regard, the existing resources developed in the Global Antimicrobial Resistance Surveillance System (2015), including national and regional networks, capacity building tools, protocols, softwares, platforms etc…, might be exploited for collecting, sharing and analyzing data on phage resistance, therefore avoiding the unaffordable development and deployment of a phage specific surveillance system. On a broader front, phage therapy should be seriously considered among the global strategies to fight AMR, potentially as a part of the Global Action Plan on Antimicrobial Resistance (2015), which WHO helps lead.

## The Phage Therapy Alternative Business Model

Personalized medicinal products based on natural phages are not prone to strong patent protection and broad market distribution (Minssen, 2014; Todd, 2019). As such, phage therapy does not fit the market model that prevails for pharmaceuticals (Pirnay et al., 2011, 2012). Not surprisingly, the mainstream pharma industry has seemingly shown little interest in this area. Instead, this therapeutic practice is confined to not-for-profit phage therapy centers, hospitals or, to a lesser extent, some niche biotech companies. This limited commercial attractiveness poses a threat to the fundraising needed to meet the canonical pharmaceutical standards of quality, safety and efficacy. At the same time, however, this represents an opportunity for emerging countries, which might be motivated to include inexpensive phages in their medicinal practice. Under this scenario, WHO could be a valuable resource to compensate for the shortcomings of knowledge, technology, and regulatory skill associated with the limited incomes fueled by phage therapy. This makes the engagement of WHO, which operates as a not-for-profit international organization, positively essential for the mobilization of resources to be allocated to phage therapy deployment, especially in LMICs.

## The Regulatory Challenge for Phage Therapy

Widely distributed phage therapy medications are made available through pharmacies in Russia and Georgia. These products are approved following regulatory processes that typically apply to ready-made preparations produced at a commercial scale (Chanishvili and Sharp, 2009). However, phage therapy may be regulated differently. Because of their narrow host range, phages could be used to specifically treat a given infectious event. Based on a “phagogram” establishing the susceptibility profile of the infecting bacteria, a customized preparation could be formulated by mixing suspensions of phages shown to lyse the infectious agent—and this could be done on an individual or local scale. However, the changing content of these preparations entails serious regulatory difficulties. Indeed, virtually all regulatory frameworks are meant to license industrially-prepared medicinal products that display a fixed qualitative and quantitative composition. In contrast, patient specific pharmaceutical preparations are usually considered as magistral formulas in the EU or compounded prescription drug product in the US. This led Belgium to implement a pragmatic approach for regulating phage therapy, based on the provision of the legislation on magistral preparation (Pirnay et al., 2018).

Another hindrance relates to the manufacturing requirements of PTMPs. Whereas, the need for a quality system is not questioned here, the GMP, as currently applicable for standard industry-made medicines, are typically out of reach for potential PTMP manufacturers. Indeed, full-blown GMP for PTMPs would imply major investments, out of proportion with the very small-scale production of named patient therapeutic phages. Instead, an *ad hoc* quality standard should be laid down, which ensures both the quality of the product and the safety for the patient, while allowing local productions at an affordable price. Table 1 gives the minimum quality standards that should be applied in a pragmatic way to ensure that PTMPs are of suitable quality for their intended use.

**Table 1:** Summary of the quality standards provided in the Belgian Phage Active Pharmaceutical Ingredient (API) monograph (Pirnay et al., 2018).

**Process**	**Minimum quality standards**
Quality system	Phage APIs are manufactured from phage seed lots under a quality system.
Production environment	The manufacturing of phage APIs takes place in an environment with specified air quality and cleanliness to minimize the risk of contamination. The effectiveness of these measures is validated and monitored. Where phage APIs are exposed to the environment during processing, without a subsequent microbial inactivation or removal process, an air quality with particle counts and microbial colony counts equivalent to those of Grade A, as defined in the current European Guide to Good Manufacturing Practice (GMP), Annex 1 and Directive 2003/94/EC is required with, if the system is not closed, a background environment at least equivalent to GMP Grade B in terms of particles and microbial counts.
Equipment and materials	Where equipment or materials affect critical processing or storage parameters (e.g., temperature, pressure, particle counts, and microbial contamination levels), they must be identified and subjected to appropriate monitoring, alerts, alarms, and corrective action, as required, to detect malfunctions and defects and to ensure that the critical parameters are maintained within acceptable limits at all times. All equipment with a critical measuring function is calibrated against a traceable standard if available. Maintenance, servicing, cleaning, disinfection, and sanitation of all critical equipment are performed regularly and recorded accordingly. Standard Operating Procedures (SOPs) detail the specifications for all critical materials and reagents. In particular, specifications for culture media, additives (e.g., solutions) and packaging materials are defined. Where applicable, reagents and materials meet compendial requirements and/or documented specifications and the requirements of Regulation 2017/745 of the European Parliament and of the Council of 5 April 2017 on medical devices and Regulation 2017/746 of the European Parliament and of the Council of 5 April 2017 on *in vitro* diagnostic medical devices. Animal component free culture media and additives should preferably be used. Host bacteria used in the manufacturing process are as safe (or least pathogenic) as possible. Non-lysogenic bacterial strains are used, if possible.
Purification	Purification methods (e.g., filtration and affinity chromatography) need to be applied to minimize the content of harmful bacterial or culture medium components (e.g., bacterial endotoxins and animal products).
Preservation/Storage	Phage seed lots and phage APIs need to be stored using validated preservation/storage methods (e.g., cooling, cryopreservation, and freeze-drying).
Release testing (by a Belgian Approved Laboratory)	**Phage seed lots**. • **Phage identification**. State of the art DNA or RNA sequencing and genome analysis. When reliable *in silico* morphology prediction is not possible, phage morphology should be determined by electron microscopy. • **Phage enumeration**. The phage enumeration of the phage seed lot should be determined using an appropriate method (e.g., pfu determination or qPCR). • **Phage purity**. Absence of adventitious agents (e.g., other phages, bacteria, or viruses) should be demonstrated using an appropriate method, unless otherwise justified (e.g., virus testing may be omitted if no human or animal origin is used). • **Detection of genetic determinants conferring toxicity, virulence, lysogeny, and antibiotic resistance**. State of the art DNA or RNA sequencing and genome analysis.
	**Phage APIs**. All tests are performed under appropriate quality standards (e.g., ISO17025). • **Phage identification**. The phage strain of a phage API is determined using a validated or qualified phage identification test (e.g., specific PCR, qPCR). • **Quantitative assessment of phages**. The potency of the phage API is determined using a validated or qualified assay (e.g., phage-specific qPCR). • **Quantitative bioburden determination (EP 2.6.12)**. The total aerobic microbial count is determined using the official Ph.Eur. method, or where justified and authorized, using a validated alternative method. Phage APIs are required to contain ≤10 cfu/100 ml or g. • **Bacterial endotoxins (EP 2.6.14)**. The test for bacterial endotoxins is used to detect and quantify endotoxins of gram-negative bacterial origin using amoebocyte lysate from horseshoe crab (*Limulus polyphemus* or *Tachypleus tridentatus*). The endotoxin limit depends on the final therapeutic product (magistral preparation) and its route of administration and is stated in the individual monograph according to compendial requirements. The maximal dose administered by the intended route per hour should not contain sufficient endotoxin to cause a toxic reaction. For instance, as stated in EP 5.1.10, the maximum dose for intravenous injection is 5 Endotoxin Units (EU)/kg/h. • **Potentiometric determination of pH (EP 2.2.3)**. The pH should conform to the pH specifications set forth in the individual monograph, usually 6.0–8.0 pH. • **Water content (EP 2.5.12 or 2.5.32)**. Dried phage APIs are tested for water content. The maximum water content is 3.0 per cent m/m, unless otherwise stated in specific monograph (e.g., APIs intended for oral lyophilisates). • **Impurities**. Process-related impurities should be quantified and qualified. Appropriate acceptance criteria should be set up such that the amounts of impurity intake are consistently below levels that are demonstrated to be safe.
Shelf life	Phage quantity (using a stability indicative method), bioburden, pH and, where relevant, water content are periodically determined. The shelf life is the time period during which phage quantity, bioburden, pH, and where relevant water content, of the API remain within the specified limit thresholds.
Labeling	The label states all information necessary to identify the content and specific instructions or warnings for administration, storage and disposal (e.g., phage identity and quantity, host bacteria, storage conditions, the production date, the expiration date, instructions for reporting serious adverse reactions and/or events, and instructions how to dispose of unused (expired) products).
Surveillance	The clinical use of phage API based magistral preparations must be surveyed and reported, including possible adverse events and reactions associated with their use. A centralized reporting system and a register for therapeutic phage applications are warranted.

In contrast to the prescriptive, strict, and sometimes dogmatic application of GMP standards by national authorities, the GMP audits conducted by WHO follow a more realistic approach. The patient is central in this exercise and there is no compromise on his or her safety and on the quality requirements of medicines. However, the WHO approach may take the form of a partnership aimed at strengthening the quality standards of the manufacturers rather than an authoritative inspection. Similar principles could be applied to the manufacture of PTMPs and instead of requiring strict adherence to standard GMP, *ad hoc* “Sound Manufacturing Practices” might be implemented for phages under the auspices of WHO.

## WHO Prequalification as a Tool for Assuring Quality of Phage Stocks

In 1987, WHO launched the vaccines prequalification (PQ) programme. PQ was conceived as a service provided by WHO to United Nations (UN) procurement agencies such as UNICEF, which, following completion of the tender process, will eventually select or “qualify” the bidder(s) (Dellepiane and Wood, 2013). Initially, PQ was limited to the testing of vaccine lots, review of summary lot protocols, and inspection of manufacturing facilities. Today, PQ has become a hallmark of quality and trust, which has been expanded in various ways. First, the scope of vaccines PQ has been reinforced since it now includes a scientific review of quality, safety, and efficacy evidences, and the participation of the national regulatory authority (NRA) of the vaccine manufacturing country. Second, the PQ process has been extended to other products and services such as medicines, active pharmaceutical ingredients (APIs), *in vitro* diagnostics, and vector control products. Third, the PQ process broadened its customer base. Indeed, whereas PQ was initially intended to UN procurement agencies, today, national governments, international organizations, public-private partnerships and NGOs can also make use of this service. Finally, it is worth mentioning that PQ goes beyond a strict evaluation exercise since it provides technical assistance to product manufacturers, training, capacity building and benchmarking of NRA, advisory activities to stakeholders, as well as opportunities for collaboration between regulators.

In contrast to NRAs, WHO is not a regulatory authority. As such, it is not restrained by burdensome lawmaking processes and, consequently, it can enjoy some flexibility in the implementation of procedures, especially to address critical global health issues. The PQ of vector control products nicely illustrates WHO's capability to implement a procedural response for controlling products that are otherwise not regulated. It is precisely the situation that PTMPs are facing today. Phage therapy, as a promising response against antimicrobial resistance, offers an evident public health potential. However, because of the legal conundrum it elicits, only a handful of countries set up a regulatory framework for PTMPs. Unquestionably, a WHO PQ of phage stocks would provide a major boost to the controlled and safe use of phage therapy. Also, phage PQ would particularly benefit countries with poor regulatory oversight, which are also those that most urgently need PTMPs.

Since the regulatory requirements of phage therapy are expected to primarily focus on the phage stocks (and not on the finished product), WHO PQ of APIs appears as a particularly relevant model. Typically, PQ programmes aim at assessing the suitability of chemical APIs for use in the manufacture of finished pharmaceutical products (FPP) (WHO, Essential Medicines and Health Products: Prequalification of Medicines^1^. It involves evaluation of data relating to their quality, as well as inspection of the relevant manufacturing site(s). This service is intended for FPP manufacturers, which can then rely on potential sources of good quality APIs manufactured in compliance with GMP. This scheme could easily be transposed to the PQ of phage stocks, making sure that the responsible pharmacist is confident about the quality of the stocks to be used as active ingredients for the formulation of PTMP.

## Vertical Cooperation as a Must for Successful Phage Therapy Deployment

Phage therapy can only realize its full potential through the implementation of vertical synergies involving local, national and global stakeholders.

Phage therapy is intrinsically best served by local applications, for at least three reasons. First, there is a greater chance of finding an active phage in the local environment of the bacteria that it is meant to target. Second, phage therapy should best be prescribed bedside (for individuals) or at local scale (for regional populations), following the interpretation of a phage susceptibility test of the infecting bacteria (phagogram), ideally following specialist microbiologist advice (Henein, 2013). Third, bacterial infections usually require a quick response: the closer to the phage stock, the faster the response. As such, it makes much sense to produce PTMPs close to their point of use. This perfectly fits with the initiative of six United Nations agencies and international organizations, including WHO, which co-signed the Interagency Statement on Promoting Local Production of Medicines Other Health Technologies (2019). During the launch event held in Geneva on 24 May 2019, the Director-General of WHO, Dr. Tedros Adhanom Ghebreyesus, said that “Local production has obvious benefits for health by creating a reliable and affordable supply of essential medicines and other health products… It also creates jobs and contributes to economic growth… but realizing the promise of local production is not straightforward” (World Health Organization, 2019). Indeed, as reported in the accompanying press statement, “local production of quality-assured health products requires a holistic approach that considers policy coherence, regulatory systems strengthening, access to finance for sustainable production, a careful assessment of the business case, development of skilled human resources, access to technology for production and needs-based innovation, creation of investment incentives, and other factors, to enable manufacturers to comply with international quality standards, be competitive and engage in sustainable manufacturing” (UNAIDS, 2019). Whereas, part of this endeavor lies in the hands of national authorities, all these achievements cannot be fulfilled by local governments alone, especially since in many LMICs the capacity of both local manufacturers to produce and supply quality medical products and the NRAs to ensure quality, efficacy, and safety are insufficient. This is where the involvement of international organizations could make a difference. Considering that the manufacturing technology of phages is neither cutting-edge nor expensive, that these therapeutics follow a non-traditional business model and that, ideally, they should be produced *in situ*, PTMPs emerge as a model for sustainable and qualitative local productions of medicines, as envisioned and promoted by WHO.

Phage therapy also requires global action. Whereas, the physical management of the phage seed stocks and collections should remain in the hands of local practitioners, the phage information (targeted bacterial species/strains/isolates, genotypic, and phenotypic characterization, production host strains, etc.) should be gathered in a publicly available central database for at least two reasons. First, ensuring open access to such information would enable quick response to patients' needs—and timely response can save the lives of those with bacterial infections. Second, establishing an exhaustive sequence database would make it possible to implement a ground-breaking and extremely promising approach to phage therapy. In this cutting-edge process, phage genome sequences, and their phenotypic characteristics (i.e., target bacteria) could be analyzed by deep learning algorithms to instantly establish a match between the infecting bacteria identified bedside and the curative phage. In the future, based on the matching phage sequence available in the database, the corresponding phage genome could even be synthesized locally in order to produce a synthetic phage on site, which could be swiftly administered to the patient. Collecting the data of phage and matching bacterial genome sequences from a large number of laboratories would be greatly enhanced if it takes place under the auspices of a major and financially disinterested public health player, which inspires confidence and assures long term stability (Pirnay, 2020), in accordance with the terms of the Nagoya protocol. WHO rises up as a reliable supportive guarantor of such an undertaking.

Additionally, surveillance and pharmacovigilance information should be reported centrally and integrated in this database. Where applicable, phage stocks' “labeling” information should be updated with the safety and efficacy information collected through pharmacovigilance monitoring.

More generally, a global health strategy should be implemented to avoid the past mistakes, such as those related to the unconsidered use of antibiotics. Since the administration of phages as antimicrobial agents might result in acquired resistance, integrated use policy, responsible management, as well as good practices and guidelines should be implemented globally.

Phage therapy implementation thus requires global, national and local initiatives. As such, WHO is particularly well-placed to drive this process. Being an international organization, WHO is in a position to implement health practices at a global level. Thanks to its network of regional and country offices and several decades of interaction with NRAs, WHO may also exert its influence at both national and local levels.

## To Conclude

Implementation of phage therapy is accompanied by considerable difficulties, but in the face of the 10 million people who will likely die annually by 2050 due to AMR, public health policy makers cannot afford to exclude promising tools in the struggle against infections caused by multidrug resistant bacteria (O'Neil, 2014; De Vos and Pirnay, 2015). WHO is challenged to play a key role in this process, which would primarily benefit LMICs. WHO possesses the medical, scientific and programmatic capabilities to meet this challenge, and, with the PQ program, it already harbors a powerful procedural tool, which might suitably regulate this unorthodox therapeutic practice.

It is our hope that this call will be heard.

## Author Contributions

AF conceived of the overall topic and defined the contents of the manuscript. AF, TN, CF, and J-PP wrote the text. GV, DD, and MM reviewed the manuscript. All authors read and approved the final manuscript.

## Conflict of Interest

The authors declare that the research was conducted in the absence of any commercial or financial relationships that could be construed as a potential conflict of interest.

## Footnotes

^1^https://extranet.who.int/prequal/content/active-pharmaceutical-ingredients-0
